# Adsorption of Ten Microcystin Congeners to Common Laboratory-Ware Is Solvent and Surface Dependent

**DOI:** 10.3390/toxins9040129

**Published:** 2017-04-06

**Authors:** Stefan Altaner, Jonathan Puddick, Susanna A. Wood, Daniel R. Dietrich

**Affiliations:** 1 Human and Environmental Toxicology, University of Konstanz, P.O. Box 662, 78457 Konstanz, Germany; Stefan.altaner@uni-konstanz.de; 2 Cawthron Institute, Private Bag 2, Nelson 7010, New Zealand; jonathan.puddick@cawthron.org.nz; 3 Environmental Research Institute, University of Waikato, Private Bag 3105, Hamilton 3240, New Zealand; susie.wood@cawthron.org.nz

**Keywords:** acidification, cyanobacteria, cyanotoxin, glass, liquid chromatography-tandem mass spectrometry, polypropylene, sample handling, sample loss

## Abstract

Cyanobacteria can produce heptapetides called microcystins (MC) which are harmful to humans due to their ability to inhibit cellular protein phosphatases. Quantitation of these toxins can be hampered by their adsorption to common laboratory-ware during sample processing and analysis. Because of their structural diversity (>100 congeners) and different physico-chemical properties, they vary in their adsorption to surfaces. In this study, the adsorption of ten different MC congeners (encompassing non-arginated to doubly-arginated congeners) to common laboratory-ware was assessed using different solvent combinations. Sample handling steps were mimicked with glass and polypropylene pipettes and vials with increasing methanol concentrations at two pH levels, before analysis by liquid chromatography-tandem mass spectrometry. We demonstrated that MC adsorb to polypropylene surfaces irrespective of pH. After eight successive pipet actions using polypropylene tips ca. 20% of the MC were lost to the surface material, which increased to 25%–40% when solutions were acidified. The observed loss was alleviated by changing the methanol (MeOH) concentration in the final solvent. The required MeOH concentration varied depending on which congener was present. Microcystins only adsorbed to glass pipettes (loss up to 30% after eight pipet actions) when in acidified aqueous solutions. The latter appeared largely dependent on the presence of ionizable groups, such as arginine residues.

## 1. Introduction

**Figure 1:**
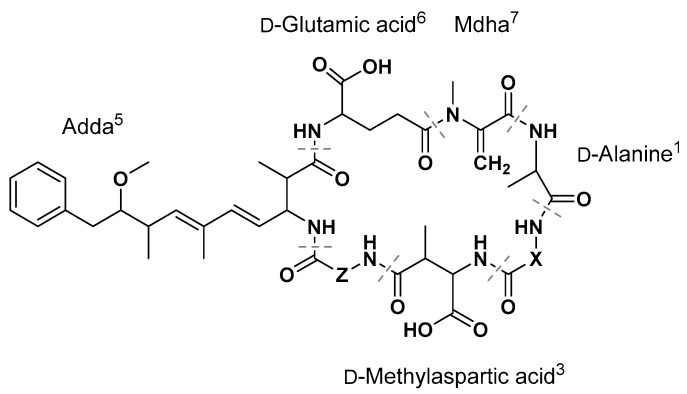
General structure of a microcystin. The structure contains the unusual amino acids Adda ((2*S*,3*S*,8*S*,9*S*,4*E*,6*E*)-3-amino-9-methoxy-2,6,8-trimethyl-10-phenyl-4,6-decadienoic acid) and Mdha (*N*-methyldehydroalanine), as well as (l-) and (d-) amino acids.

Microcystins (MC) are a family of structurally related heptapeptides, produced by some freshwater cyanobacteria. They are potent inhibitors of cellular serine/threonine-protein-phosphatases (ser/thr-PP) [1]. Consequently, when humans and animals are exposed (mainly via ingestion), the liver is primarily affected due to the first-pass effect and the expression of organic anion transporting polypeptides (OATPs) [2]. Toxicodynamics are characterized by irreversible ser/thr-PP inhibition and subsequent protein hyper-phosphorylation leading to loss of cell structure, apoptosis, and necrosis [3,4]. Acute intoxications result in human morbidity and occasional mortality [5], while chronic exposure is associated with increased incidences of liver tumors [6,7]. Structurally, MC consist of proteinogenic (l-) and non-proteinogenic (d-) amino acids with a generalized sequence of cyclo-[d-Ala]-[X]-[d-Masp]-[Z]-[Adda]-[d-Glu]-[Mdha] (Figure 1). Here, [X] and [Z] are variable l-amino acids, Mdha is *N*-methyldehydroalanine, and d-Masp is d-methylaspartic acid. The amino acid Adda, (2*S*,3*S*,8*S*,9*S*,4*E*,6*E*)-3-amino-9-methoxy-2,6,8-trimethyl-10-phenyl-4,6-decadienoic acid, is a unique amino acid, a priori only observed in MC and the related nodularins [8]. Along with the two variable positions X and Z, structural differences in the congeners arise from variable methylation/demethylation patterns (reviewed in [9]). The amino acids in the X- and Z-positions define the name of the respective congener, for example, MC-LR has leucine (L) in the X-position and arginine (R) in the Z-position (Figure 1, Table 1). To date, more than 100 congeners have been identified [10]. This high number of congeners makes analysis challenging, as physicochemical parameters, such as hydrophobicity or ion formation, differ according to the functional groups of the amino acids in the variable positions.

Eutrophication and rising temperatures have been associated with increased mass occurrences (blooms) of toxin-producing cyanobacteria globally [11,12]. Consequently, and in conjunction with an ever increasing world population and enhanced use of water per capita [13,14], the risk for human intoxication by MC is also increasing. Thus, reliable analysis (identification and quantification) of these toxins is critical to sustain safe drinking and recreational waters. Potential hazard and exposure assessment currently employs the congener addition concept, whereby all microcystin congeners present in a sample are expressed as MC-LR equivalents (reviewed in [15]). However, the latter approach not only wrongly assumes that all microcystins have the same toxicokinetics and dynamics [16,17,18], but also presumes that all congeners behave similarly during sample processing and handling. Indeed, previous analyses have demonstrated that losses of different congeners can occur during sample handling [19,20,21,22]. Thus, it is crucial to analyze all congeners present in a certain sample, without loss due to pre-analysis handling. Losses during handling can happen during transfer steps or storage through adsorption to commonly used surfaces like polypropylene or various kinds of glass material.

Studies have shown that MC adsorption to plastic-ware was dependent on the solvent used (i.e., methanol or acetonitrile concentration) and differed according to the structural characteristics of the MC congeners [19,20,21]. The pH of the solvent can affect adsorption, with acidified methanol leading to less adsorption of MC to GF/C filters, which are commonly used during sample preparation, compared to non-acidified solvents [22]. 

However, currently there are no uniform suggestions on how to handle MC-containing samples and solutions. Microcystin congeners might behave differently in various solvents, depending on the amino acids present in positions X and Z. Due to the presence of certain functional groups, such as carboxyl groups or guanidine moieties, in the variable positions of the MC structure, differences in hydrophobicity could affect the extent of MC loss. In the present study, the influence of acidification and methanol (MeOH) concentration on the loss of ten structurally-diverse MC congeners to polypropylene and glass surfaces was assessed.

## 2. Results

### 2.1. Adsorption of Microcystin Congeners to Polypropylene Pipette Tips in Aqueous and High-Percentage Methanol Solutions

A structurally-diverse range of MC congeners including di-arginated, mono-arginated, and non-arginated variants were used to assess the effect of solvent composition on MC adsorption to polypropylene laboratory-ware. MCs were dissolved in either water, 80% methanol, or acidified versions (0.1% formic acid added, pH around 2.7) thereof and distributed into identical LC-MS/MS vials for each MC congener–solvent combination. While one set served as a control (assumed 100% MC recovery) the others were used to investigate adsorption to the pipette tips during pipetting (1, 2, 4, or 8 pipette aspirations and releases). 

**Figure 2:**
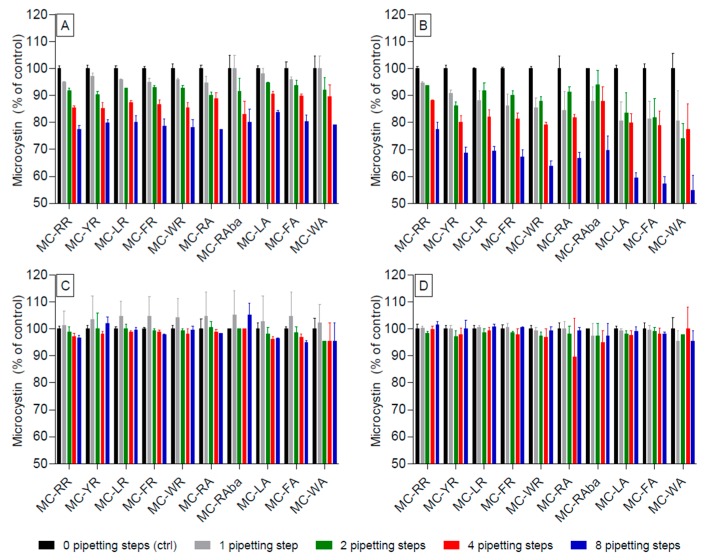
Reduction of various microcystin (MC) congeners in acidified and non-acidified solvents after increasing steps of pipetting using polypropylene pipette tips. MC were spiked into water (**A**); acidified water (**B**); 80% methanol (**C**); or acidified 80% methanol (**D**); and subjected to increasing numbers of pipetting steps. Acidification was achieved using 0.1% formic acid. Significance levels are not inserted in the graph for clarity reasons, but are available in Table S1.

When the MC congeners were dissolved in non-acidified water, no statistically significant loss due to adsorption was observed after one pipetting step (Figure 2A, Table S1). However, two pipetting steps resulted in a significant (*p* < 0.05) adsorption (10% of total) for all congeners (except for MC-LA). Similarly, a loss of ca. 20% of their original concentration (*p* < 0.001, Figure 2A, Table S1) was observed after four and eight pipetting steps for all MC congeners. 

A significant (*p* < 0.01) loss of >5% resulted for all MC congeners, except for MC-RR and MC-YR, when they were dissolved in acidified water (pH = ~2.7) already after one pipetting action. Most MC congeners encountered further losses due to adsorption with increasing pipetting steps, whereby after the eighth step, the total loss for most congeners reached 40%, resulting in 60% of the concentration at the outset (Figure 2B, Table S1). In contrast to other MC congeners, MC-RR showed a significant (*p* < 0.01) loss to pipette adsorption only after four (and eight) pipetting steps. Overall, the use of acidified water resulted in approximately double the loss than observed for non-acidified aqueous solutions.

No significant reduction in MC concentration was observed with one to eight pipetting actions when MC were dissolved in non-acidified 80% methanol, (Figure 2C). This was also apparent when the MC were dissolved in 80% methanol acidified with 0.1% FA (Figure 2D). Within these experiments, MC-RA when four pipetting were used (Figure 2D), showed surprisingly large data variability (large standard deviation). The latter was tested for outliers using the Grubb’s test, which indicated that one of the raw data points could be an outlier, an interpretation that is supported by the fact that all other values for MC-RA, irrespective of the number of pipetting steps, did not differ significantly from the 100% control.

**Figure 3:**
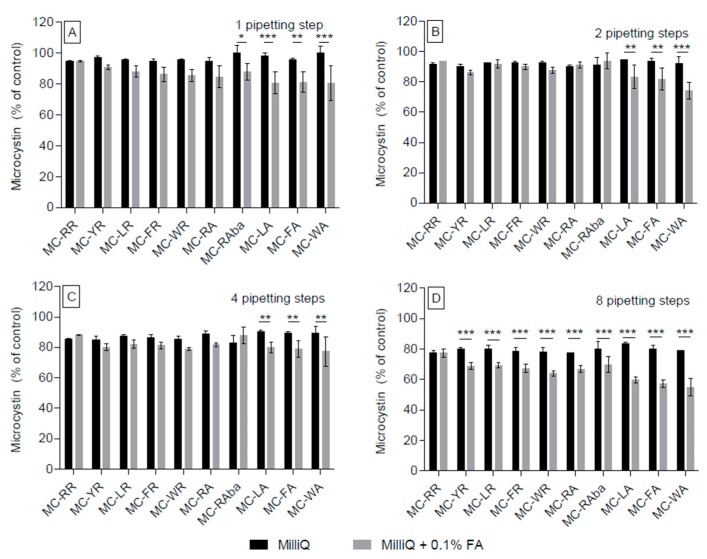
Comparison of water and acidified water for the individual repeats of successive pipetting steps. The data used for Figure 2 was reanalysed comparing only water (black bars) and acidified water (grey bars) directly with each other following additional pipetting steps, one (**A**); two (**B**); four (**C**); and eight additional steps (**D**). * *p* < 0.05, ** *p* < 0.01, *** *p* < 0.001.

The side-by-side comparison of aqueous and acidified aqueous solutions (Figure 3) moreover demonstrated that non-arginine-containing congeners experienced significant adsorption loss in the acidified solution already after the first 1–4 pipetting steps (*p* < 0.01, Figure 3A–C), whereas this was the case for all MC congeners (except for MC-RR) after eight pipetting steps (Figure 3D).

### 2.2. Effect of Methanol Concentration on the Adsorption of Selected Microcystins to Polypropylene Pipette Tips

**Figure 4:**
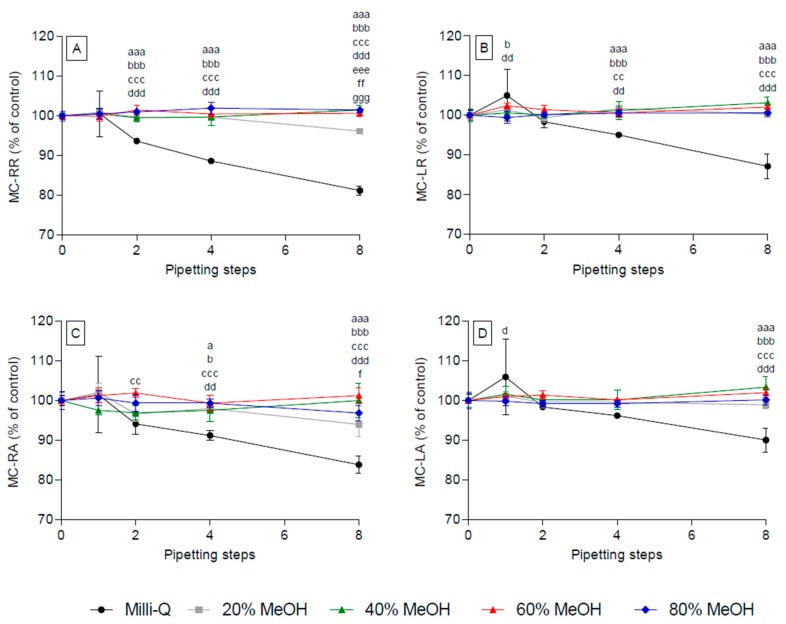
Effect of methanol concentration on the adsorption of selected microcystins to polypropylene pipette tips. Individual samples were subjected to eight successive pipetting steps. Representative congeners (double-, single-, non-arginated) include MC-RR (**A**); MC-LR (**B**); MC-RA (**C**); and MC-LA (**D**). Controls were performed without using polypropylene pipette tips (0 pipetting steps) in triplicates. Small letters represent significance levels at the individual pipetting steps: a, water vs. 20% MeOH; b, water vs. 40% MeOH; c, water vs. 60% MeOH; d, water vs. 80% MeOH; e, 20% MeOH vs. 40% MeOH; f, 20% MeOH vs. 60% MeOH; g, 20% MeOH vs. 80% MeOH. Significance levels are represented by the repetition of the letters, e.g., a, *p* < 0.05; aa, *p* < 0.01; aaa, *p* < 0.001.

Adsorption of MC-RR, -FR, -WR, -RAba, and -RA was completely avoided in aqueous solutions containing ≥40% MeOH, whereas an adsorption loss was observed after eight pipetting steps in solutions containing 20% MeOH (Figure 4A/C and Figure S1). Similarly, all other congeners tested showed a lack of adsorption in all solutions containing MeOH, irrespective of the number of pipetting steps (Figure 4, Figure S1).

### 2.3. Effect of Acidified Methanol Concentration on the Adsorption of Selected Microcystins (MC) to Polypropylene Pipette Tips

**Figure 5:**
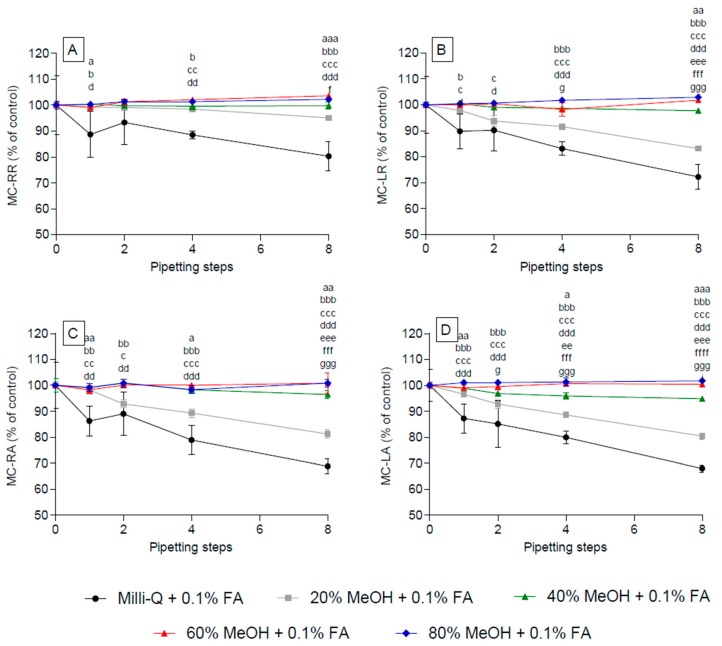
Effect of acidified methanol concentration on the adsorption of selected microcystins to polypropylene pipette tips. Individual samples were subjected to eight successive pipetting steps. Representative congeners (double-, single-, non-arginated) include MC-RR (**A**); MC-LR (**B**); MC-RA (**C**); and MC-LA (**D**). Solvents were acidified by adding 0.1% (*v*/*v*) of formic acid (FA) to the individual solvents. Controls were performed without using polypropylene pipette tips (0 pipetting steps) in triplicate. Small letters represent the significance levels at the individual pipetting steps: a, water vs. 20% MeOH; b, water vs. 40% MeOH; c, water vs. 60% MeOH; d, water vs. 80% MeOH; e, 20% MeOH vs. 40% MeOH; f, 20% MeOH vs. 60% MeOH; g, 20% MeOH vs. 80% MeOH. Significance levels are represented by the repetition of the letters, e.g., a, *p* < 0.05; aa, *p* < 0.01; aaa, *p* < 0.001.

Acidification of aqueous and aqueous-MeOH solutions resulted in increasing adsorption of all MC congeners with increasing pipetting steps, except for the doubly-arginated MC-RR which showed no decrease in 20% MeOH (Figure 5, Figure S2). However, for all congeners no adsorption was observed when the MeOH concentrations were increased to ≥40%.

### 2.4. Adsorption of Selected Microcystins (MC) in Acidified and Non-Acidified Aqueous Solutions to Glass-Ware (Pasteur Pipettes)

**Figure 6:**
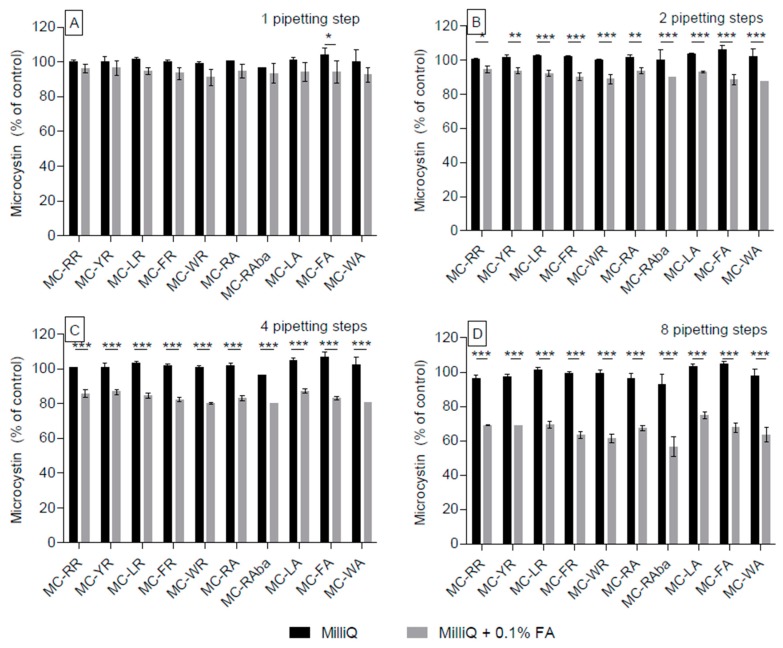
Multiple pipetting action using glass (Pasteur) pipettes. Microcystin (MC) amounts in water (water, black bars) and acidified water (water + 0.1% Formic Acid (FA), grey bars) after one (**A**); two (**B**); four (**C**); and eight steps (**D**) of pipetting actions using glass (Pasteur) pipettes. * *p* < 0.05, ** *p* < 0.01, *** *p* < 0.001.

No adsorption was observed in non-acidified aqueous solutions, irrespective of the number of pipetting steps with glass pipettes (Figure 6, Figure S3A). However, similar to the observations in polypropylene pipette tips, acidification of the aqueous solutions resulted in a significant adsorption of all MC congeners observed at ≥2 pipetting steps with glass pipettes (Figure 6B–D). The degree of adsorption appeared more pronounced with glass than with polypropylene pipettes (Figure 3 and Figure 6).

### 2.5. Effect of Acidified Methanol Concentration on the Adsorption of Selected Microcystins to Glass-Ware (Pasteur Pipettes)

**Figure 7:**
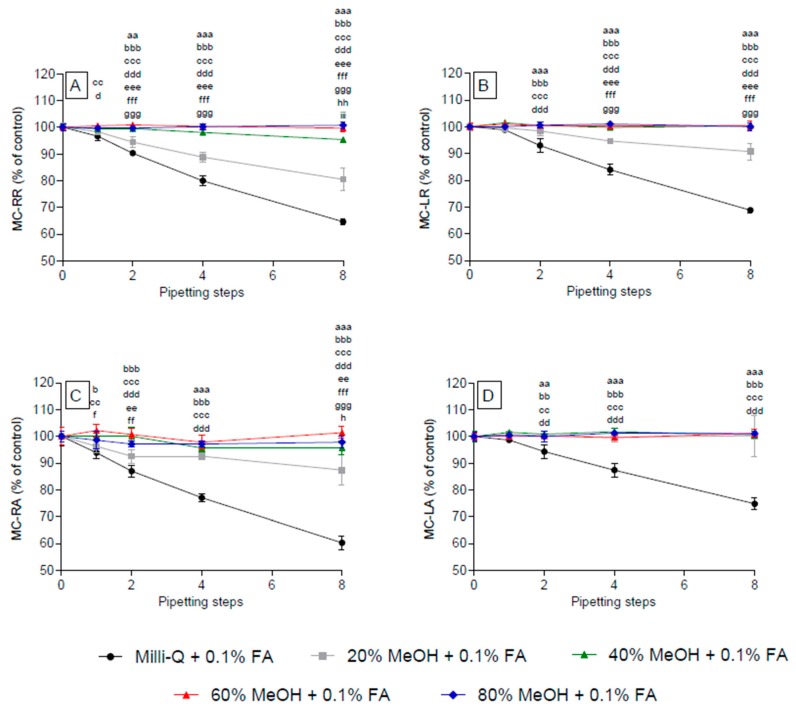
Effect of acidified methanol concentration on the adsorption of selected microcystins to glass-ware (Pasteur pipettes). Individual samples were subjected to eight successive pipetting steps. Representative congeners (double-, single-, non-arginated) include MC-RR (**A**); MC-LR (**B**); MC-RA (**C**); and MC-LA (**D**). Solvents were acidified by adding 0.1% (*v*/*v*) of formic acid (FA) to the individual solvents. Controls were performed without additional pipetting steps after distribution to the sample vials. Small letters represent significance levels at the individual pipetting steps: a, water vs. 20% MeOH; b, water vs. 40% MeOH; c, water vs. 60% MeOH; d, water vs. 80% MeOH; e, 20% MeOH vs. 40% MeOH; f, 20% MeOH vs. 60% MeOH; g, 20% MeOH vs. 80% MeOH; h, 40% MeOH vs. 60% MeOH. Significance levels are represented by the repetition of the letters, e.g., a, *p* < 0.05; aa, *p* < 0.01; aaa, *p* < 0.001.

As non-acidified aqueous solutions resulted in no detectable adsorption of the ten MC congeners tested (Figure S3 and Table S2), the question was raised whether acidification would increase the adsorption of MC congeners even in solutions containing MeOH. Indeed, as expected, acidified solutions containing no MeOH resulted in increasing adsorption with increasing number of pipetting steps, whereby this adsorption was alleviated by the addition of MeOH to the solutions (Figure 7). Of interest, however, was the finding that arginine containing MC congeners appeared also affected by the acidification of aqueous solutions even when 20% MeOH was added, whereas this was not the case for MC congeners without arginine residues, e.g., MC-LA (Figure 7, Figure S4).

### 2.6. Short Term Storage of MC Solutions in Glass or Polypropylene Vials

The comparison between glass or polypropylene vials demonstrated no difference in congener adsorption (Figure S5) after 2 h, irrespective of whether the solutions were acidified or not. However, the addition of MeOH to the aqueous solution resulted in lower adsorption of all congeners when compared to the aqueous solution without MeOH, suggesting that the use of MeOH will result in lowered losses during the analytical process.

## 3. Discussion

Microcystins are known to be present in different charge states according to the pH of the environment they are in [23,24,25], and the different ionizable functional groups they possess. Most MC congeners have two carboxylic groups (on Masp^2^ and Glu^6^, Table 1) and zero to two guanidine groups, depending on whether there are arginine residues present in the two variable positions X and Z. The fact that acidification of the solution (whether water or MeOH-water mixtures) resulted in an increased loss of MC congeners, primarily in glass but also to some extent in polypropylene pipettes, and is most likely due to different ionization states of the MC congeners at different pH. Indeed, the differences observed appear pronounced between MC congeners containing no, one, or two arginine residues (Table 2). At very low pH, MC are likely to be positively charged, as carboxyl groups and guanidine groups, if present, are protonated [23,24,25]. With increasing pH, the carboxylic groups initially become deprotonated yielding neutral (MC-RR) or single-negatively charged compounds (MC-XRs), followed later by the guanidine groups, resulting in double-negatively charged compounds in basic solutions. MC congeners not containing arginine residues most likely are double-negatively charged at lower pH values than arginated ones. At which pH MC are present in a neutral state or single charged (+ or −) state is not easily discerned. The calculated pKa values of MC-RR, MC-LR, and MC-YR are all similar and lie around pKa 3.5 [25]. For non-arginated congeners, no pKa values are known, but they could be higher as there is no ionizable guanidine group present.

The adsorption to polypropylene surfaces can be seen similar to the mechanism of reversed-phase chromatography. In this case, the polypropylene surface of the used pipette tip resembles the stationary phase, to which analytes may adhere and therefore are not able to be analyzed via liquid chromatography-tandem mass spectrometry (LC-MS/MS), as they are no longer present in solution. The solvent used resembles the elution solution (mobile phase). The higher the elution strength of a solvent towards the respective MC congener, the more likely it will stay in solution, rather than adhering to the pipette tips. To successfully retain the MC congeners in solution, the polarity of the solvent must be decreased. Water has a polarity index around twice as high as methanol (10.2 vs. 5.1) [26]. By adding methanol to water the polarity of the solution decreases and provides the MC congener with an environment which is more favorable than the surface of the pipette tip.

In acidic environments the polarity of MC changes. After acidification (the pH of 0.1% FA is ca. 2.7), MC are expected to be ionized due to the deprotonation of their carboxylic functions (pKa ca. 3.5 [25]). It is difficult to explain the higher retention to polypropylene in this situation, but an ion-pair retention mechanism could be causing the increased adsorption in acidic solutions [25]. Negatively charged ions (here: formate, HCO_2_^−^) would interact with positively charged guanidine groups forming uncharged complexes. These subsequently may interact with the polypropylene (“stationary phase”) more favorably and require higher methanol concentrations to replace them. Additionally, the guanidine groups may also interact with carboxyl groups (from the same MC molecule or from another one in proximity) again forming complexes which are uncharged, thus increasing their adsorption to the uncharged polypropylene surface. 

Corroborating observations from previous studies [19,20,21], the lipophilicity/hypdrophilicity of the MC congeners (Table 2) in our study also appeared to be governed by their adsorptivity to non-charged pipetting material, especially as MC congeners dissolved in water, and adsorbed to polypropylene laboratory-ware increasingly with the number of pipetting steps. The microscopic structure of the pipette tips used in this study (Axygen, Maxymum Recovery) is a relatively smooth surface, as protruding polypropylene chains are removed using acid treatment by the manufacturer. Because of this, the uncharged hydrophobic MC congeners might adhere to this smooth uncharged surface due to hydrophobic interactions.

During the adsorption to glass surfaces, the acidity/alkalinity of the surface may play a major role. Glass consists of glass formers (SiO_2_, B_2_O_3_, P_2_O_5_, etc.), glass modifiers (Na_2_O, K_2_O, CaO, etc.), and intermediates (BeO, MgO, Al_2_O_3_, etc.). Generally speaking, the formers are Lewis acids, the modifiers are Lewis bases, and the intermediates are amphoteric [27]. As glass formers are the major part of (soda lime) glass, the surface is most likely negatively charged in aqueous solutions, as dissociation occurs at the silanol groups [28]. In acidic environments, as used during the present study (around pH 2.7), the silanol groups are most likely still in an ionic (negative) state, as they would require extremely low pH to become protonated [29]. Therefore, the increased adsorption which was observed in acidic solvents, most likely occurred through the differential ionization of the MC congeners as opposed to that of the glass surface. As discussed earlier, in acidic environments MC are likely to be present in protonated form. As the surface of the soda-lime glass is negatively charged and MC are positively charged in acidic environments, interactions between silanol groups of the glass surface and the guanidine group of MC are most likely leading to increased adsorption.

A previous study [22] observed an effect from solvent acidification on the amounts of MC binding to GF/C filters (glass-fiber, class C). The researchers found that arginine-containing MC adhered to the GF/C material when in neutral methanol. In acidified methanol, the adsorption was absent for singly-arginated MC and only partially reduced for MC-RR. When we tested the effects of solvent acidification on MC adsorption to soda-lime glass (as opposed to the borosilicate glass used in GF/C filters), the MC adsorption was enhanced in acidified water. However, MC adsorption to soda-lime glass was not observed in non-acidified water or methanol solutions. When acidified solutions were supplemented with 40% methanol, the loss of MC was negated.

Heussner et al. observed no loss of arginine-containing MC congeners when using borosilicate glass vials as storage containers for MC [19]. In their study, solutions were stored in 5% non-acidified methanol and there was no control using an acidified storage solution. The researchers assessed the recovery after up to two months and did not observe any losses of MC-RR, MC-LR, or MC-YR, but losses were noted for MC-LA, MC-LF, and MC-LW. In light of the results presented here, it might be possible that Heussner et al. did not actually see loss due to storage in glass, but due to the repeated contact with polypropylene tips during sample preparation for the ELISA analysis used to measure MC in the study. When using ELISA detection for MC, the maximum amount of methanol in the final solution is less than 5%. This may have led to the apparent loss of non-arginated congeners but not arginine-containing MC. In our study, short-term storage of MC congeners in solution with low methanol concentrations (0%–40%) did not differ when polypropylene or glass vials were used.

A recent study also using ELISA reported that MC-LR, MC-LA, and MC-LF adsorb to various surfaces like polypropylene, polystyrene, high-density polyethylene, and polycarbonate when stored up to 120 h [30]. The amount of absorbed toxin was dependent on the type of water the toxins were in. The authors saw the highest adsorption in deionized water, followed by chlorine-quenched drinking water and surface water. The quenching of chlorine in the drinking water was crucial, as MC were rapidly degraded without quenching. Only glass and polyethylene terephthalate could stop adsorption effectively from the water types tested. As all materials of the storage containers were compared to glass and not to absolute recovery, it cannot be excluded that loss occurred (through the pipetting steps necessary for the ELISA). It is also possible that the quenching did not actually lead to degradation, but altered the polarity of the present congeners and increased their adsorptive behavior.

Collectively, our results and those from previous studies show that under non-acidified conditions, 5% methanol is sufficient to limit the loss of arginated MC congeners to polypropylene surfaces, but at least 40% methanol should be used, if possible, to reduce the loss of other (non-arginated) congeners. The abundance of non-arginated congeners present in environmental samples might be higher than reported to-date, as they are more easily lost during sample preparation procedures.

## 4. Conclusions

In the present study, MC congeners were shown to adsorb to two common materials used during laboratory handling of samples: polypropylene and soda-lime glass. The level of adsorption was dependent on the structure and thus the physico-chemical properties of the MC congeners, the acidity/polarity of the solvent in which the MC were dissolved, and how many pipetting actions were performed. Microcystin congeners dissolved in water adsorbed to polypropylene and this was more severe when the solutions were acidified. The adsorption of MC to soda-lime glass was only apparent in acidified solutions. Under acidic conditions, the number of guanidine moieties (in the arginine residues) in the individual congeners is critical for the extent of adsorption (Table 2). Addition of methanol rectifies the observed loss.

In order to avoid adsorption when working with MC-containing solutions, it is recommended that a methanol concentration of ≥40% and a neutral pH is used. The latter recommendation differed from those provided in previous studies [19,31,32,33]. Of importance for researchers working with MC congeners is, to recognize the fact that congeners differ in their ability to be ionized. The latter is a critical determinant for their polarity, behavior in solution, and thus adsorption. In consequence, disregarding the physico-chemical properties of MC congeners and thus not using the appropriate solvents will result in massively different recoveries of the original sample concentrations and therefore possibly to analytical results misrepresenting the true situation in the sample.

## 5. Materials and Methods

### 5.1. Reagents and Laboratory-Ware

The Milli-Q water used was deionized water with 18.2 MΩ purified using a Milli-Q Plus ultra-pure water system still (Millipore, Billerica, MA, USA). Formic acid (FA; Merck, Kenilworth, NJ, USA) and acetonitrile (Honeywell, Morris Plains, NJ, USA) were of MS grade. Analytical standards for MC-RR, MC-LR, and MC-YR were from DHI LAB products (Hoersholm, Denmark). Liquid Chromatography vials were made from clear glass or polypropylene with lids having pre-split septa (Phenomenex; Torrance, CA, USA).

### 5.2. Production of Microcystin Congener Stock Solution

A lyophilised extract of *Microcystis* CAWBG11, containing di-arginated, mono-arginated, and non-arginated MC congeners [34], was dissolved in water. For a working stock, the extract was diluted in water to a nominal concentration of 100 ng/mL of MC-RR whilst other congeners ranged from 6.5 ng/mL (MC-RAba) to 314.5 ng/mL (MC-LR). Only the results for congeners MC-RR, -YR, -LR, -FR, -WR, -RA, -RAba, -LA, -FA, and -WA are presented. These were chosen as they were present in sufficient amounts to be reliably quantified with the UPLC-MS/MS method used. Additional congeners present in the extract (desmethyl and didesmethyl variants of MC-RR and MC-LR and Aba containing variants of MC-LA, -FA, and -WA) were also detected, but the amounts were too low to reliably assess the loss of those congeners to adsorption. In general, we observed that these modified congeners followed a similar adsorption pattern as their basic chemical entity (e.g., desmethyl-MC-LR showed a similar trend as MC-LR).

### 5.3. Adsorption of Microcystins to Common Pipetting Laboratory-Ware in Non-Acidified and Acidified Solvents

The MC working stock was diluted 1:4 with solutions of differing methanol concentrations (0%, 25%, 50%, 75%, and 100%) which were either neutral or acidified with 0.1% FA resulting in solutions with 0%, 20%, 40%, 60%, and 80% methanol.

Using glass Pasteur pipettes, the different MC solutions were distributed into the individual glass vials containing 0.3 mL inserts, preparing triplicates for each solution (volume 0.2 mL). Afterwards the individual samples were subjected to either 0, 1, 2, 4, or 8 pipetting actions using a 200 µL auto-pipettor with polypropylene pipette tips (AXYGTR-222-C-L; Axygen, Corning, NY, USA) or glass Pasteur pipettes. A new pipette tip/Pasteur pipette was used for each pipetting action to ensure no saturation of the tip binding capacities. The solutions were then analyzed by UPLC-MS/MS to determine the MC concentration. Loss due to adsorption to the pipette tips was defined as the difference in concentrations found between the control and the resulting MC concentration in the solvent after pipetting.

### 5.4. Short-Term Storage in Glass or Polypropylene Vials

The MC working stock was again diluted 1:4 into the same matrices as above to achieve a nominal concentration of 20 ng/mL MC-RR. The solution was distributed into the individual samples in either glass LC-vials (Verex AR0-3010-13; Phenomenex) or polypropylene LC-vials (Verex AR0-9994-13-C; Phenomenex) using a three times equilibrated (take in and blow out of solution) polypropylene pipette tip. A sample volume of 600 µL was used in the glass vials and 512 µL for polypropylene vials. The different volumes were used to ensure that the solutions cover the same surface area in the respective vials (calculated from the inner diameter of the respective vials). Vials were then incubated for 1 h at 100 rpm on a rotator to mimic sample handling and stored at 4 °C until analyses (around 1 h later).

### 5.5. Ultra-performance liquid Chromatography-Tandem Mass Spectrometry (UPLC-MS/MS) Detection of Microcystins

For MC analyses, an Acquity I-class liquid chromatograph (Waters, Milford, MA., USA) equipped with a Waters Acquity BEH C_18_ column (1.6 µm, 2.1 × 50 mm) coupled to a XEVO TQ-S mass spectrometer was used (Waters). Column temperature was set to 40 °C. The used solvents were A: 10% acetonitrile + 100 mM FA and 6 mM ammonia; B: 90% acetonitrile + 100 mM FA and 6 mM ammonia. The used gradient was the same as published previously [35]. The injected sample volume was 5 µL.

The used masses and transitions for the investigated congeners can be found in Table 3. Compounds entering the mass spectrometer were ionized using a capillary voltage of 1.5 kV and a nebulizer pressure of 7.0 bar. For desolvation, a nitrogen flow of 1000 L/h at 500 °C was used.

### 5.6. Outlier Analysis

Outlier analysis was performed by two-sided Grubb’s test using an online tool provided on the homepage of GraphPad Prism software at a significance level of *p* < 0.05 [36]. In order to define outlier(s) and to determine their potential effects on the results and interpretation, data were statistically analyzed (see below) with and without the inclusion of the presumed outlier(s). 

### 5.7. Data Handling and Statistical Analyses

Concentrations of MC were quantified using MassLynx 4.1 software (Waters). Microsoft Excel was used to process the data of the individual samples. Means and standard deviations (SD) were generated from the triplicates. Means were normalized to control values, which were set as 100%. Percentile standard deviation was calculated by normalizing standard deviations to the mean of the control. Statistical analyses were performed using GraphPad Prism 5 software (GraphPad Software, Inc., La Jolla, CA, USA). Statistical tests were either a 2-way-ANOVA with Bonferroni post-test, or a Student’s *t*-test and were indicated in the figure legends of the individual graphs or tables. Means ± SD of triplicate analyses were depicted. 

## Figures and Tables

**Table 1:** Microcystin congeners subjected to the experimental procedures described here.

MC	X^2^	Z^4^
MC-RR	l-Arginine	l-Arginine
MC-YR	l-Tyrosine	l-Arginine
MC-LR	l-Leucine	l-Arginine
MC-FR	l-Phenylalanine	l-Arginine
MC-WR	l-Tryptophan	l-Arginine
MC-RA	l-Arginine	l-Alanine
MC-RAba	l-Arginine	l-Aminobutyric acid
MC-LA	l-Leucine	l-Alanine
MC-FA	l-Phenylalanine	l-Alanine
MC-WA	l-Tryptophan	l-Alanine

The amino acids in the X^2^ and Z^4^ positions denote the abbreviated name for the individual congeners. For example MC-LR has l-Leucine in position X^2^ and l-Arginine in position Z^4^.

**Table 2:** Percentage of methanol needed to counteract the loss of microcystins from acidic and non-acidic solutions.

Microcystin Variant	Non-Acidified		Acidified	
	Glass	Polypropylene	Glass	Polypropylene
Doubly-arginated (more hydrophilic)	0 (Milli-Q water)	20	40	20
Singly-arginated (amphiphilic)	0 (Milli-Q water)	40	20	40
Non-arginated (more lipophilic)	0 (Milli-Q water)	40	20	40

**Table 3:** Mass spectrometric parameters of the used microcystins (MC).

Congener	Parent	Daughter	Cone Voltage	Collision Energy
	(m/z)	(m/z)	(V)	(V)
MC-RR	519.7	135.1	40	27
MC-YR	1045.5	135.1	40	70
MC-LR	995.5	135.1	40	65
MC-FR	1029.5	135.1	40	65
MC-WR	1068.5	135.1	40	65
MC-RA	953.5	135.1	40	65
MC-RAba	967.5	135.1	40	65
MC-LA	910.6	135.1	40	65
MC-FA	944.6	135.1	40	65
MC-WA	983.6	135.1	40	65

## Supplementary Materials

The following are available online at www.mdpi.com/2072-6651/9/4/129/s1, Figure S1: Reduction of all investigated microcystin (MC) congeners in solutions with decreasing methanol (MeOH) concentrations due to adsorption to polypropylene; Figure S2: Reduction of all investigated microcystin (MC) congeners in solutions with decreasing acidic methanol concentrations using polypropylene pipet tips; Figure S3: Reduction of various microcystin congeners in acidified and non-acidified solvents after increasing steps of pipetting using Pasteur pipettes; Figure S4: Reduction of all investigated microcystin (MC) congeners in solutions with decreasing acidic methanol (MeOH) concentrations using Pasteur pipettes; Figure S5: Effect of short term storage of microcystins in glass or polypropylene vials, Table S1: Significance levels for Figure 2; Table S2: Significance Levels for Figure S3.
