# Supplementary material for: Adsorption of Ten Microcystin Congeners to Common Laboratory-Ware Is Solvent and Surface Dependent
Source: Toxins (Basel). 2017 Apr 6;9(4):129. doi: 10.3390/toxins9040129 (PMC5408203; doi:10.3390/toxins9040129)
Supplement: Supplementary file 1 [file toxins-09-00129-s001.pdf]

# Supplementary Materials: Adsorption of Ten Microcystin Congeners to Common Laboratory-Ware Is Solvent and Surface Dependent

**Stefan Altaner, Jonathan Puddick, Susanna A. Wood and Daniel R. Dietrich**

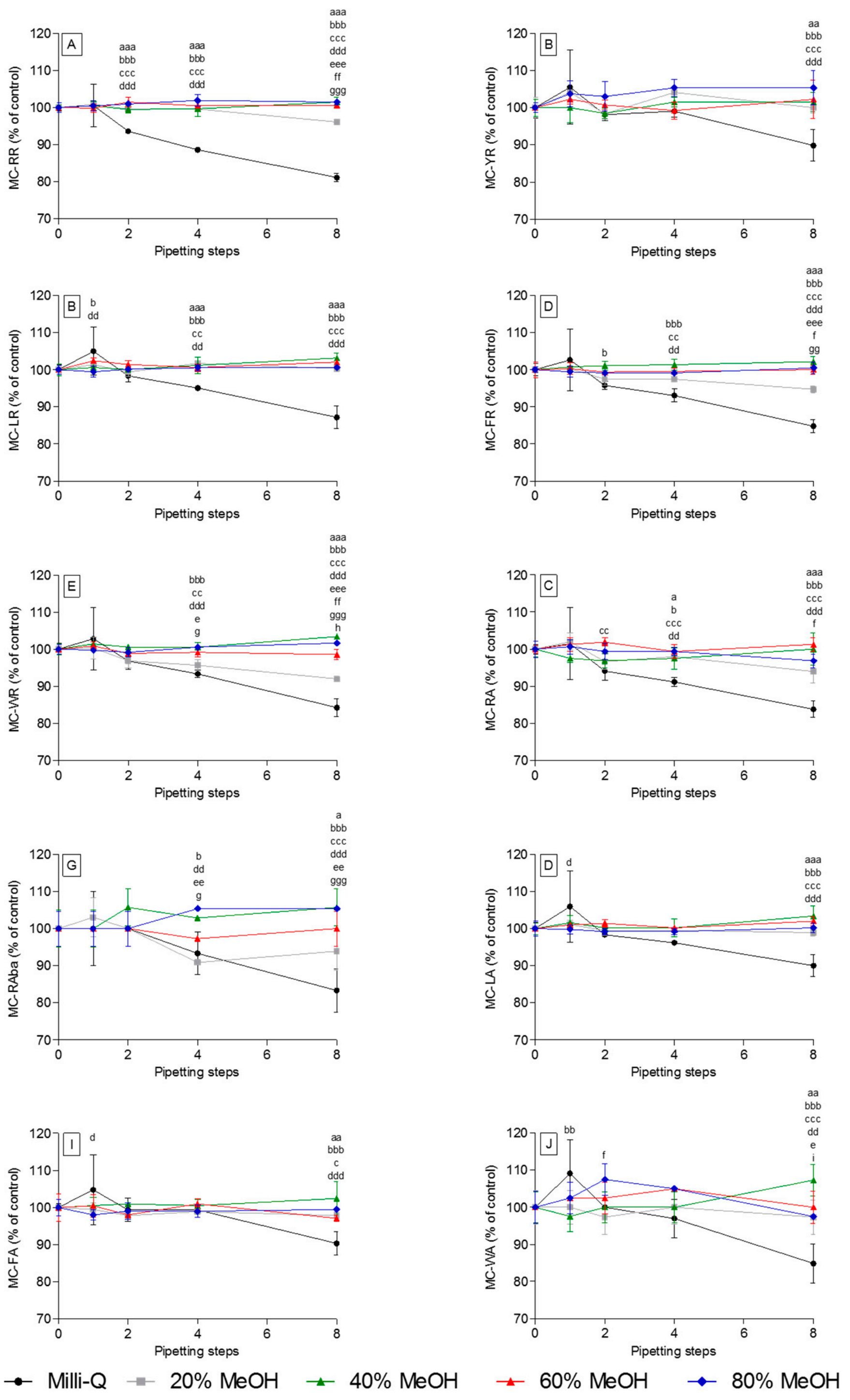


**Figure S1.** Reduction of all investigated microcystin (MC) congeners in solutions with decreasing methanol (MeOH) concentrations due to adsorption to polypropylene. Individual samples were subjected to eight successive pipetting steps. **A**: MC-RR, **B**: MC-YR, **C**: MC-LR, **D**: MC-FR, **E**: MC-WR,

**F**: MC-RA, **G**: MC-RAba, **H**: MC-LA, **I**: MC-FA, **J**: MC-WA. Controls were performed without using polypropylene pipette tips in triplicates. Small letters represent significance levels at the individual pipetting steps: a, water *vs.* 20% MeOH; b, water *vs.* 40% MeOH; c, water *vs.* 60% MeOH; d, water *vs.* 80% MeOH; e, 20% MeOH *vs.*40% MeOH; f, 20% MeOH *vs.* 60% MeOH; g, 20% MeOH *vs.*80% MeOH; h, 40% MeOH *vs.* 60% MeOH; i, 40% MeOH *vs.* 80% MeOH. Significance levels are represented by repetition of the letters, e.g.: a, $p < 0.05$; aa, $p < 0.01$; aaa, $p < 0.001$.

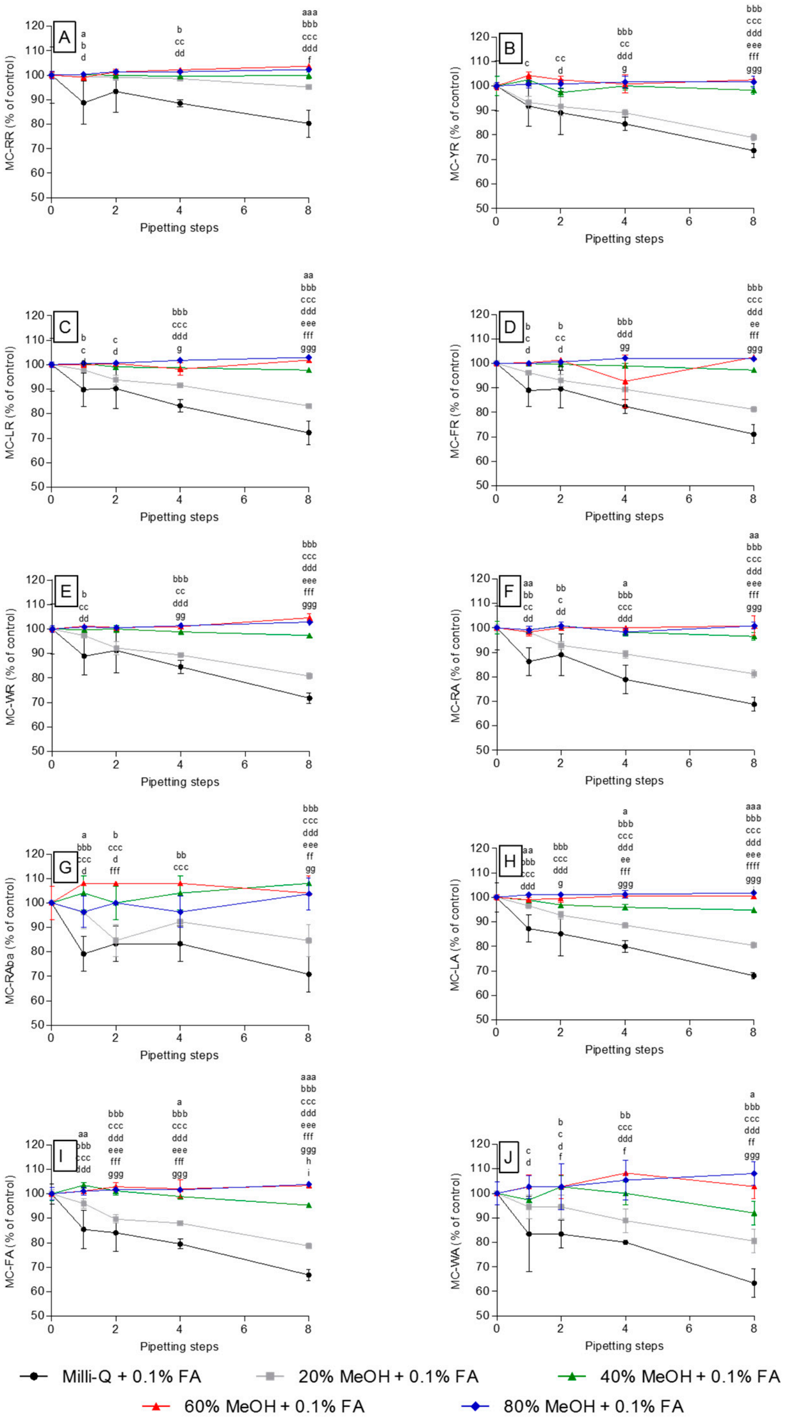


**Figure S2.** Reduction of all investigated microcystin (MC) congeners in solutions with decreasing acidic methanol concentrations using polypropylene pipet tips. Individual samples were subjected to eight successive pipetting steps. **A**: MC-RR, **B**: MC-YR, **C**: MC-LR, **D**: MC-FR, **E**: MC-WR, **F**: MC-RA, **G**: MC-RAba, **H**: MC-LA, **I**: MC-FA, **J**: MC-WA. Controls were performed without using polypropylene pipette tips in triplicates. Small letters represent significance levels at the individual pipetting steps: a, water *vs.*

20% MeOH; b, water *vs.* 40% MeOH; c, water *vs.* 60% MeOH; d, water *vs.* 80% MeOH; e, 20% MeOH *vs.*40% MeOH; f, 20% MeOH *vs.* 60% MeOH; g, 20% MeOH *vs.*80% MeOH; h, 40% MeOH *vs.* 60% MeOH; i, 40% MeOH *vs.* 80% MeOH. Significance levels are represented by repetition of the letters, e.g.: a, $p < 0.05$; aa, $p < 0.01$; aaa, $p < 0.001$.

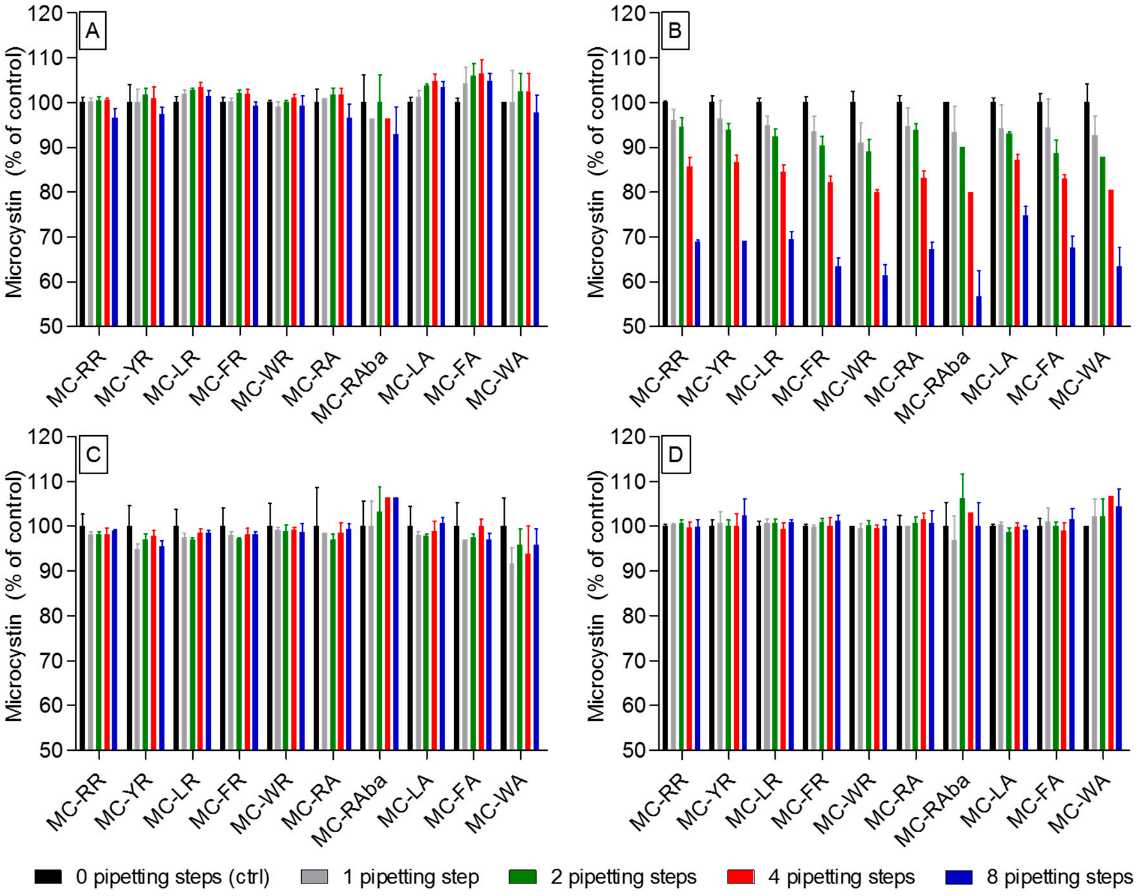


**Figure S3.** Reduction of various microcystin congeners in acidified and non-acidified solvents after increasing steps of pipetting using Pasteur pipettes. Microcystins were spiked into different solvents and submitted to the indicated pipetting steps. **A**: water; **B**: water + 0.1% formic acid, **C**: 80% methanol, **D**: 80% methanol + 0.01% formic acid. Significance levels are not inserted in the graph for clarity reasons but can be found in Table S2.

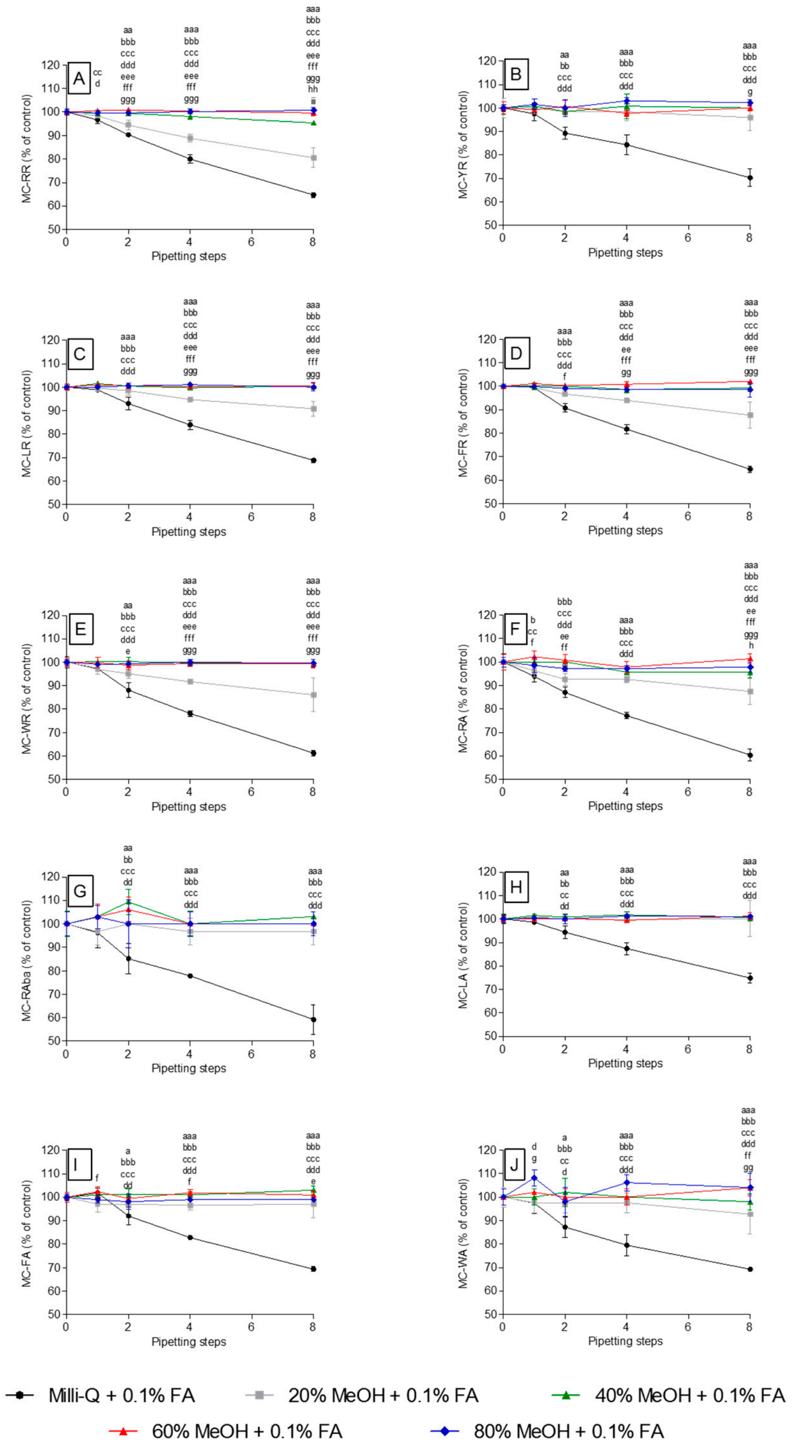


**Figure S4.** Reduction of all investigated microcystin (MC) congeners in solutions with decreasing acidic methanol (MeOH) concentrations using Pasteur pipettes. Individual samples were subjected to eight successive pipetting steps. **A**: MC-RR, **B**: MC-YR, **C**: MC-LR, **D**: MC-FR, **E**: MC-WR, **F**: MC-RA, **G**: MC-RAba, **H**: MC-LA, **I**: MC-FA, **J**: MC-WA. Controls were performed without using polypropylene pipette tips in triplicates. Small letters represent significance levels at the individual pipetting steps: a, water *vs.* 20% MeOH; b, water *vs.* 40% MeOH; c, water *vs.* 60% MeOH; d, water *vs.* 80% MeOH; e, 20% MeOH *vs.*40% MeOH; f, 20% MeOH *vs.* 60% MeOH; g, 20% MeOH *vs.*80% MeOH; h, 40% MeOH *vs.* 60% MeOH; i, 40% MeOH *vs.* 80% MeOH. Significance levels are represented by repetition of the letters, e.g.: a, $p < 0.05$; aa, $p < 0.01$; aaa, $p < 0.001$. FA, formic acid.

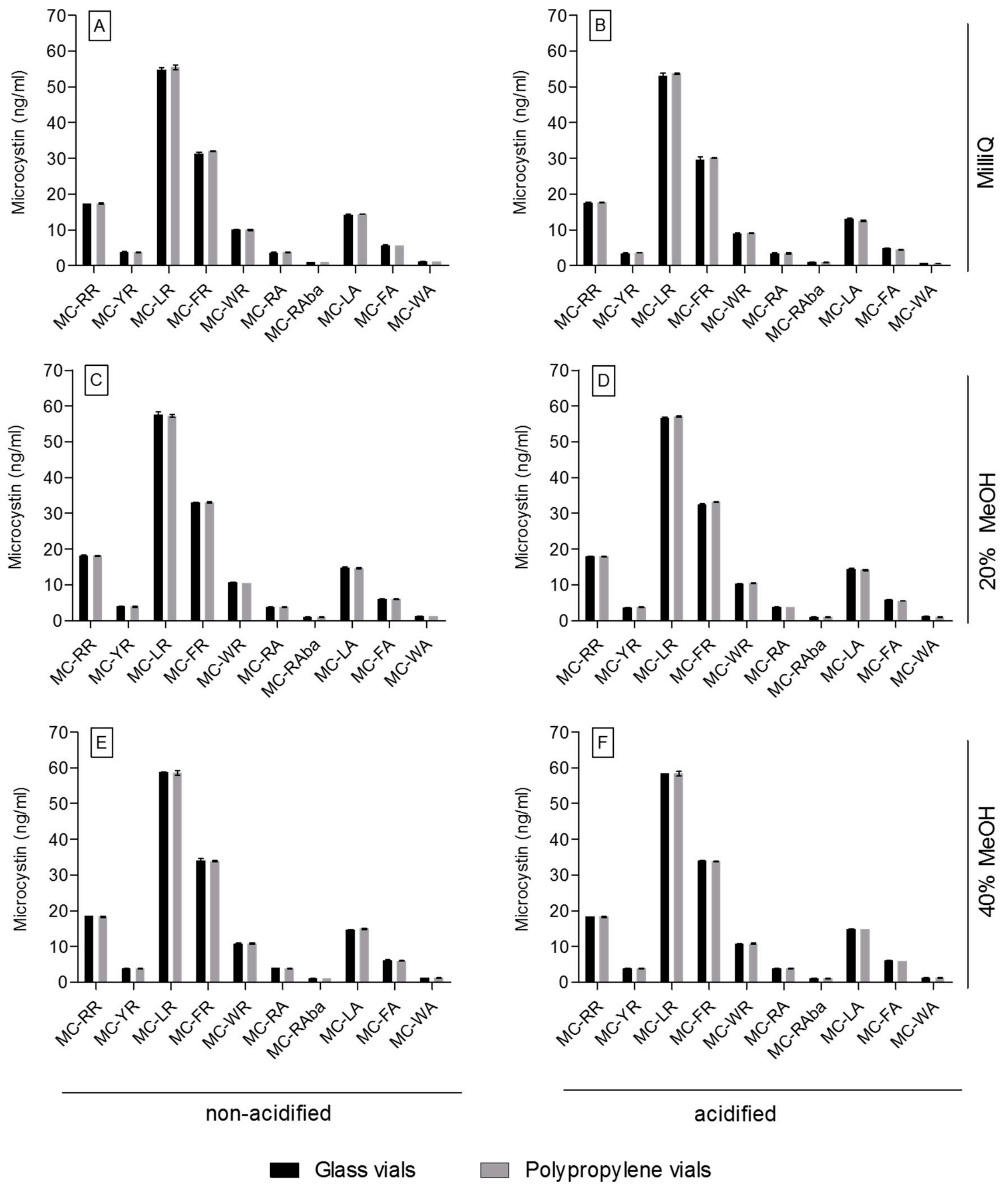


**Figure S5.** Effect of short term storage of microcystins in glass or polypropylene vials. Microcystins were spiked into non-acidified (**A**, **C**, **E**) and acidified (**B**, **D**, **F**) water (A+B), 20% methanol (C+D), 40% methanol (E+F) and distributed to either glass (black bars) or polypropylene LC vials (grey bars) before short term storage (~2 h). * $p < 0.05$, ** $p < 0.01$, *** $p < 0.001$.

**Table S1: Significance levels for Figure 2.**

| | Congener | MC-RR | | MC-YR | | MC-LR | | MC-FR | | MC-WR | | MC-RA | | MC-RAba | | MC-LA | | MC-FA | | MC-WA | |
|---|---|---|---|---|---|---|---|---|---|---|---|---|---|---|---|---|---|---|---|---|---|
| | **Experiment** | **A** | **B** | **A** | **B** | **A** | **B** | **A** | **B** | **A** | **B** | **A** | **B** | **A** | **B** | **A** | **B** | **A** | **B** | **A** | **B** |
| **Pipette actions** | **1** | - | - | - | - | - | ** | - | *** | - | *** | - | *** | - | ** | - | *** | - | *** | - | *** |
| | **2** | *** | - | *** | ** | ** | - | ** | ** | ** | ** | *** | - | *** | - | - | *** | * | *** | ** | *** |
| | **4** | *** | ** | *** | *** | *** | *** | *** | *** | *** | *** | *** | *** | *** | ** | *** | *** | *** | *** | *** | *** |
| | **8** | *** | *** | *** | *** | *** | *** | *** | *** | *** | *** | *** | *** | *** | *** | *** | *** | *** | *** | *** | *** |

A (water) and B (water + 0.1% v/v formic acid) correspond to the individual graphs of Figure 2. Experiments C (80% MeOH) and D (80% MeOH + 0.1% v/v formic acid) are not shown, as there were no significant differences to controls detected. Percentages of microcystins (MC) after the individual repetitions of pipetting steps are compared to the control (0 pipette actions). * $p < 0.05$, ** $p < 0.01$, *** $p < 0.001$, - no significance.

**Table S2: Significance Levels for Figure S3.**

| | | MC-RR | | | | MC-YR | | | | MC-LR | | | | MC-FR | | | | MC-WR | | | | MC-RA | | | | MC-RAba | | | | MC-LA | | | | MC-FA | | | | MC-WA | | | |
|---|---|---|---|---|---|---|---|---|---|---|---|---|---|---|---|---|---|---|---|---|---|---|---|---|---|---|---|---|---|---|---|---|---|---|---|---|---|---|---|---|---|
| | **Experiment** | **A** | **B** | **C** | **D** | **A** | **B** | **C** | **D** | **A** | **B** | **C** | **D** | **A** | **B** | **C** | **D** | **A** | **B** | **C** | **D** | **A** | **B** | **C** | **D** | **A** | **B** | **C** | **D** | **A** | **B** | **C** | **D** | **A** | **B** | **C** | **D** | **A** | **B** | **C** | **D** |
| | **1** | - | - | - | - | - | - | - | - | - | - | - | - | - | * | - | - | - | *** | - | - | - | - | - | - | - | * | - | - | - | - | - | - | - | - | - | - | - | * | * | - |
| **Pipette** | **2** | - | - | - | - | - | - | - | - | - | ** | - | - | - | *** | - | - | - | *** | - | - | - | - | - | - | - | *** | - | * | - | * | - | - | - | *** | - | - | - | *** | - | - |
| **actions** | **3** | - | *** | - | - | - | *** | - | - | - | *** | - | - | - | *** | - | - | - | *** | - | - | - | *** | - | - | - | *** | - | - | - | *** | - | - | * | *** | - | - | - | *** | - | ** |
| | **4** | - | *** | - | - | - | *** | - | - | - | *** | - | - | - | *** | - | - | - | *** | - | - | - | *** | - | - | * | *** | - | - | - | *** | - | - | - | *** | - | - | - | *** | - | - |

A (water), B (water + 0.1% v/v formic acid), C (80% methanol) and D (80% methanol + 0.1% FA) correspond to the individual graphs of **Figure S3**. Percentages of microcystins (MC) after the individual repetitions of pipetting steps are compared to the control (0 pipette actions). * $p < 0.05$, ** $p < 0.01$, *** $p < 0.001$, - no significance.
